# KGSCS—a smart care system for elderly with geriatric chronic diseases: a knowledge graph approach

**DOI:** 10.1186/s12911-024-02472-9

**Published:** 2024-03-12

**Authors:** Aihua Li, Che Han, Xinzhu Xing, Qinyan Wei, Yuxue Chi, Fan Pu

**Affiliations:** 1 School of Management Science and Engineering, Central University of Finance and Economics, Beijing, 100080 China; 2 Beijing Academy of Science and Technology, Research Institute for Smart Aging, Beijing, 100050 China

**Keywords:** Smart care, Knowledge graph, Geriatric chronic disease

## Abstract

**Background:**

The increasing aging population has led to a shortage of geriatric chronic disease caregiver, resulting in inadequate care for elderly people. In this global context, many older people rely on nonprofessional family care. The credibility of existing health websites cannot meet the needs of care. Specialized health knowledge bases such as SNOMED—CT and UMLS are also difficult for nonprofessionals to use. Furthermore, professional caregiver in elderly care institutions also face difficulty caring for multiple elderly people at the same time and working handovers. As a solution, we propose a smart care system for the elderly based on a knowledge graph.

**Method:**

First, we worked with professional caregivers to design a structured questionnaire to collect more than 100 pieces of care-related information for the elderly. Then, in the proposed system, personal information, smart device data, medical knowledge, and nursing knowledge are collected and organized into a dynamic knowledge graph. The system offers report generation, question answering, risk identification and data updating services. To evaluate the effectiveness of the system, we use the expert evaluation method to score the user experience.

**Results:**

The results of the study showed that compared to existing tools (health websites, archives and expert team consultation), the system achieved a score of 8 or more for basic information, health support and Dietary information. Some secondary evaluation indicators reached 9 and 10 points. This finding suggested that the system is superior to existing tools. We also present a case study to help the reader understand the role of the system.

**Conclusion:**

The smart care system provide personalized care guidelines for nonprofessional caregivers. It also makes the job easier for institutional caregivers. In addition, the system provides great convenience for work handover.

**Supplementary Information:**

The online version contains supplementary material available at 10.1186/s12911-024-02472-9.

## Background

**Fig. 1:**
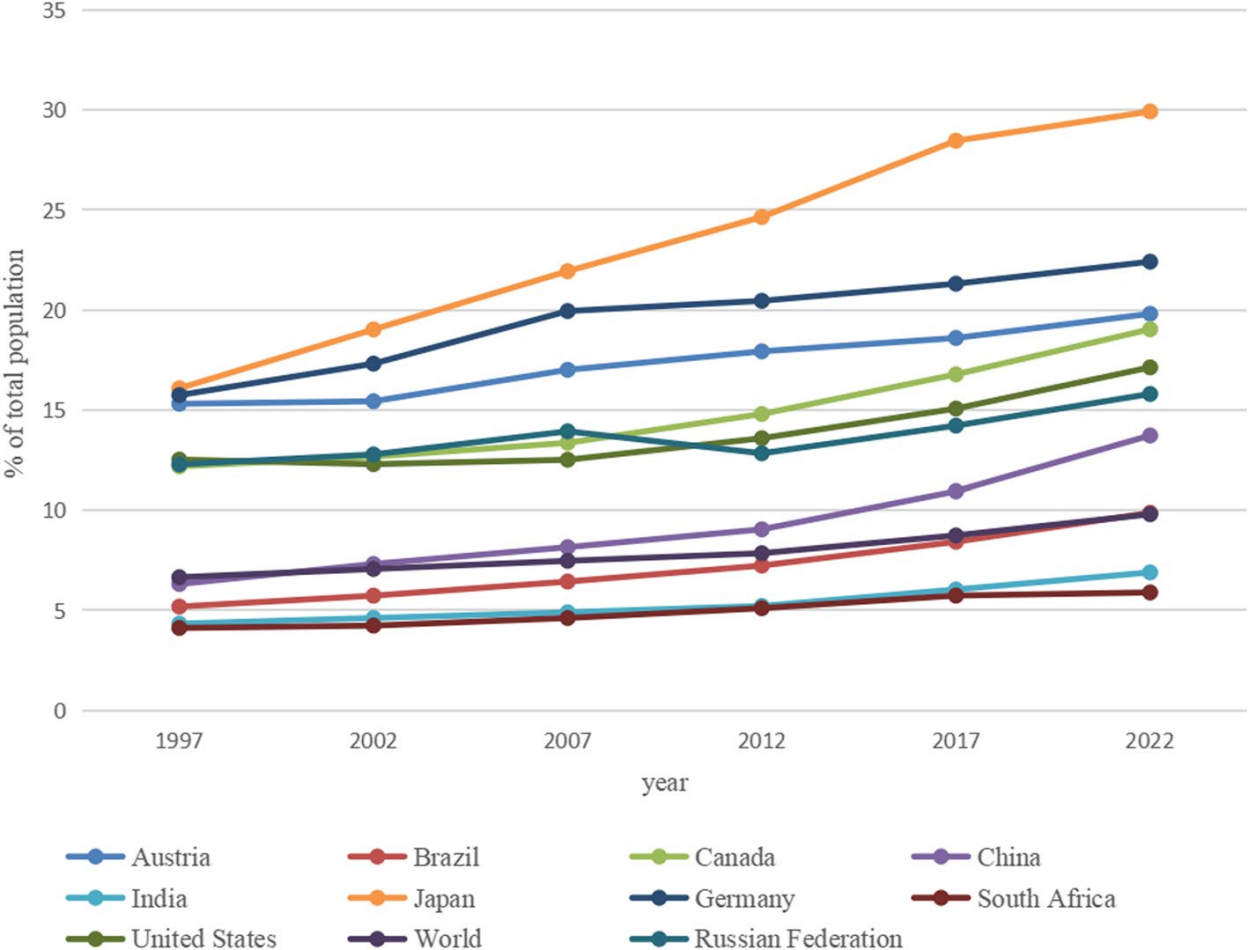
The aging situation of the world and some countries in the past 25 years

Currently, the degree of global population aging is increasing. According to statistics released by the World Bank, as of 2022, people aged older than 65 years has increased to 9.8%. In China, according to the census data from the National Bureau of Statistics, the proportion of elderly residents aged 65 and above exceeded 7.3% in 2002. By 2022, the proportion of elderly residents aged 65 and above exceeded 13.7%. China has entered a deep aging society in only 20 years, which reflects the rapid growth of China's population aging process. In Japan, 29.9% of the total population is aged 65 years and older in 2022. Aging is becoming increasingly serious worldwide, especially in developed areas (Fig. 1).

**Fig. 2:**
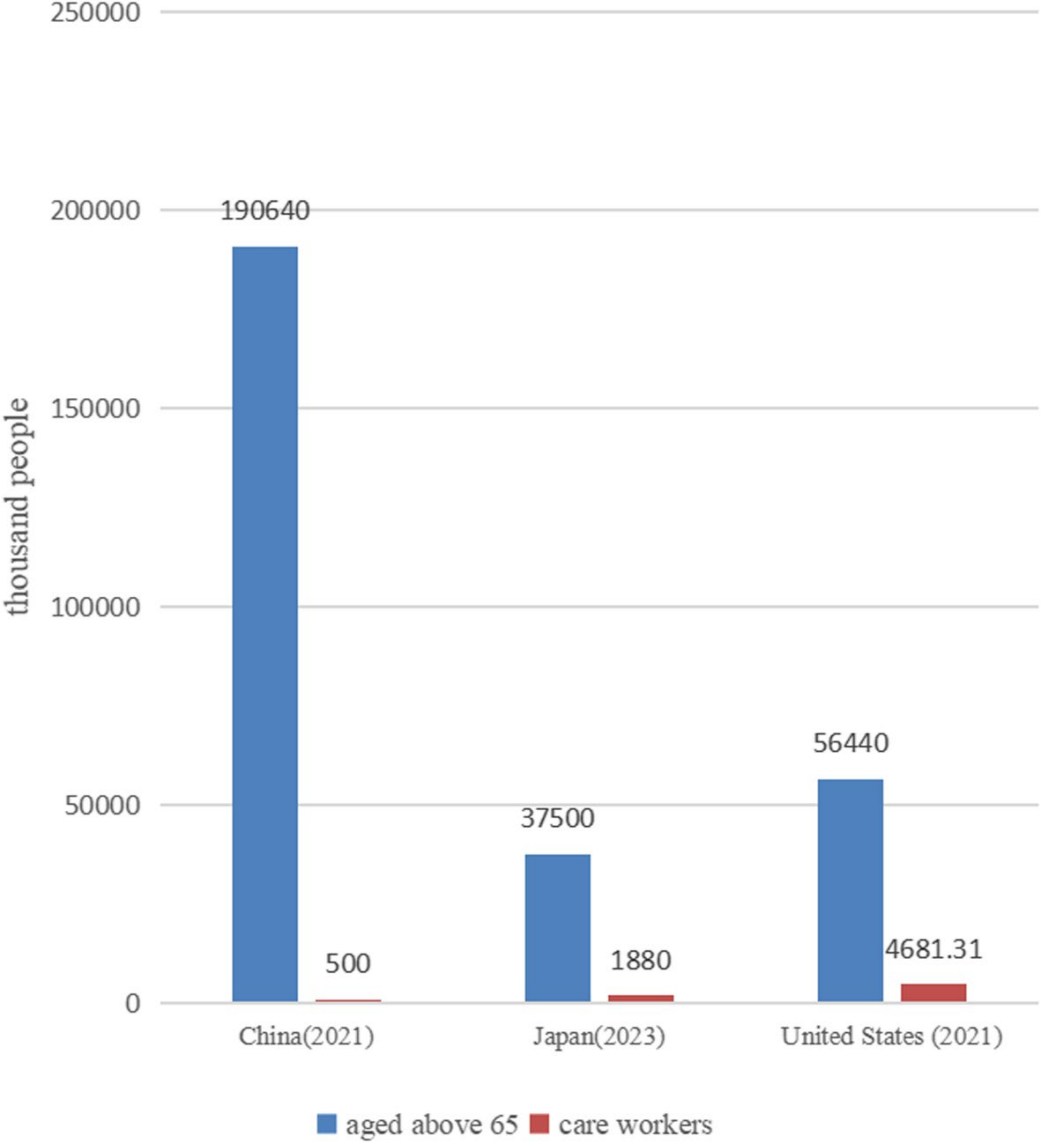
Situation of elderly care in China, Japan and United States*. *Data from China National Health Commission, Japan Ministry of Health, Labor and Welfare and United states Bureau of Labor Statistics

In this context, the demand for elderly care is increasing [1, 2]. However, the supply of care services dose not meet the demand. The shortage of caregiver and the uneven quality of care services are the greatest difficulties in the development of long-term care service systems (Fig. 2). According to the Ministry of Health, Labor and Welfare, the shortage of elderly caregivers in Japan will reach 380,000 by 2025. Some industry associations even predict that the shortage will reach 1 million. In populous China, the problem is even more severe. China needs more than 13 million caregiver [3, 4] based on the international allocation of caregiver to the disabled elderly at a ratio of 1:3. At present, the total number of caregiver in various medical and elderly care institutions is far from meeting these requirements [5]. Among them, there are fewer qualified professional caregiver to meet existing care needs. The majority of the elderly in China can only receive care from nonprofessional caregivers, such as family members or domestic helpers. For the elderly who are able to receive professional care, excess workload or replacement of caregivers can also lead to a decrease in the quality of care [6].

Nonprofessional caregivers refer to younger family members and live-in domestic helpers without professional training. Cultural variations in elderly care practices are evident, especially when comparing Western and Asian cultures. Notably, in China, the cultural importance of filial piety adds a unique dimension to elderly care. Due to a shortage of professional caregivers and traditional beliefs, elder care institutions are less favored. As a result, families often rely on younger members for elder care. When family members are busy, hiring domestic helpers becomes a prevalent choice for in-home care. Most domestic helpers are not trained caregivers but handle tasks like grocery shopping, cooking, and cleaning.

To solve the problem of low-quality care services caused by the shortage and replacement of caregiver, some studies have been conducted. Recently, the concepts of effective integrated care [7, 8], comprehensive care [9], and long-term care have gainedtraction [10]. The community hopes to provide comprehensive services such as medical, nursing and rehabilitation to the elderly to improve the efficiency of long-term care [11, 12]. In the construction of a long-term care service system, it is necessary to adopt technical means and intelligent development thinking. Therefore, the concept of smart care has emerged.

The purpose of smart care is to replace physical and mental labor in long-term care services for the elderly with chronic diseases [13, 14]. Smart care refers to a new modern care model that automatically monitors the physical health status of care objects in real time through the Internet of Things, Internet technology and various artificial intelligence technology products. Which provides comprehensive services such as life care, health care, and social support [15, 16]. The application of intelligent technology in care services is gradually promoting the transformation of traditional care services from artificial to intelligent. Smart care provides better services for the elderly, especially the disabled and semidisabled elderly.

Smart care mainly includes two aspects. One is to collect health data through smart devices to realize real-time remote monitoring of care objects [17]. It is convenient for medical institutions, communities and families to evaluate related health data [18]. The main purpose of these studies was to replace physical labor in care services. These studies include smart care devices’ auxiliary role [19], block-chain applications [20], monitoring frameworks [21], cognitive assessments [22], data interpretation issues [23], and system development [24–26].

Another aspect of the research is to establish an information platform. The platform is used as a hub for the transmission and integration of various information, human and material resources to realize the wisdom of care services [27]. The goal of the information platform is to replace or assist experts in making care plans that meet the personalized needs of care objects. The platform is used as the hub to transfer and integrate all kinds of information, human and material resources to realize the intelligence of care services [28]. Research in this area is mainly aimed at replacing mental work in care services.

For the second aspect, it is generally considered to health knowledge websites. However, health knowledge websites do not meet the needs of non-professionals. First, there are too many websites and some of them are not credible enough [29]. For nonprofessionals, it is also difficult to spot problems in time. Compared with health websites that can only be passively searched by users, professional health platforms that can actively make recommendations are more valuable.

Knowledge graphs offer a flexible and scalable framework, efficiently representing complex relationships and dependencies within diverse datasets, promoting dynamic updates, and enhancing semantic understanding for comprehensive data integration. At present, knowledge graphs are being developed rapidly and are widely used in finance, medicine and other fields [30]. The development of medical knowledge graphs has exhibited a rapid trajectory. Years of diligent research endeavors have yielded numerous generic knowledge graphs in the realm of healthcare, such as the specific medical ontologies SNOMED-CT and UMLS. The existing research has focused on the construction of disease knowledge graphs, and has little involvement in chronic disease nursing and intelligent care. Yong, Zhang [31] proposed the Health Knowledge Graph Builder (HKGB), an end-to-end platform that can be used to construct health knowledge graphs. Medical knowledge graphs are also applied in many medical fields, such as disease prediction [32], disease classification [33] and drug reuse possibility discovery [34]. However, a knowledge graph concerning chronic disease care in geriatric patients was missing. Therefore, the medical knowledge graph has many shortcomings because of the breadth of knowledge required for smart care and the need for customized information retrieval combined with individual data [35].

Due to the urgent need for smart elderly care, the shortage of smart equipment and information platform systems, and the development potential of knowledge graphs in the field of smart care, the contributions of this paper are as follows:

- *Problem*: There are enormous gaps in professional care services available for the elderly.
- *What is Already Known*: Although existing expert systems and health websites can reuse medical knowledge, these systems cannot provide personalized care service suggestions for the nonprofessional care givers.
- *What This Paper Adds*:
  1. We propose an innovative medical knowledge graph construction method to integrate multisource heterogeneous data, which include smart device data, personal information of the elderly, medical disease knowledge, and chronic disease care knowledge. Personal information and professional medical knowledge are combined organically to provide knowledge support for care givers.
  2. Personalized care guide generation, risk identification and question answering modules were constructed to construct knowledge graph based smart care system (KGSCS) for geriatric chronic diseases. The system is especially friendly to nonprofessional caregivers because it actively provide care guidance to them.
  3. After practical application and expert assessment, the KGSCS has addressed the deficiencies in everyday care service knowledge and the specific habits and preferences of elderly individuals compared to existing medical knowledge graphs such as SNOMED-CT or health websites.

The system can help professional caregivers take better care of more elderly people at the same time. At the same time, reports generated by the system can help caregivers quickly understand the situation of unfamiliar elderly people. This approach is especially convenient for the handover when there is a replacement for caregivers.

## Methods

**Fig. 3:**
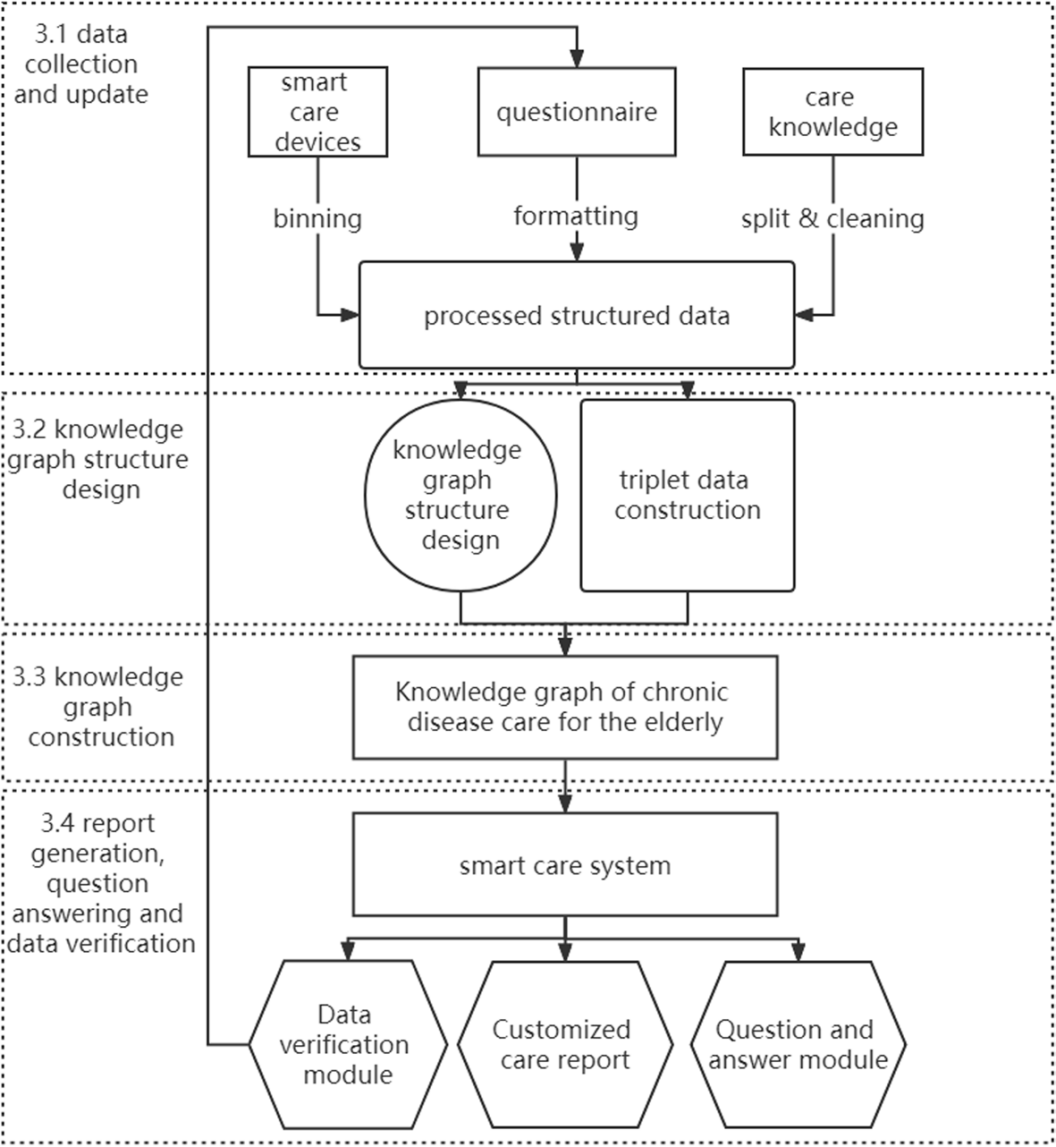
Framework of smart care system for elderly

The smart care system for chronic diseases of the elderly is designed to alleviate the lack of long-term care personnel. Based on the actual needs of elderly chronic disease care, the system needs to cover the following functions: data update, data verification, knowledge mining, information storage, information retrieval, and question answering. Therefore, the structure of the intelligent care system for elderly chronic diseases designed in this paper is as follows (Fig. 3):

### Data collection and updating

The data used in this paper are drawn from three sources. The first is the data collected by smart care devices such as smart blood pressure meters and smart mattresses. The second is a questionnaire of specific elderly people collected by their family member or caregiver, which includes more than one hundred care-related questions. The questionnaire is shown in the Supplementary material. These two data sources were collected and updated by the data platform of relevant institutions.

The data platform stores received structured questionnaire data and raw sensor data in local files. Corresponding files are updated when there are changes in the questionnaire content or updates in sensor data. For instance, when the smart blood pressure monitor generates new readings daily, the system transforms the blood pressure values into blood pressure types based on medical standards. If there is a change in blood pressure type (e.g., from normal to high blood pressure), the system updates the connections between relevant nodes in the file. It removes the connection between the elderly person's blood pressure node and the normal blood pressure node while adding a connection to the high blood pressure node.

The third is chronic disease nursing publications and open source medical knowledge. The data updating function will also facilitate the inclusion and compatibility of additional data from existing knowledge repositories. By continually updating and expanding our system, we aim to accommodate a broader spectrum of professional knowledge and ensure the longevity and relevance of our research. These data are processed for knowledge graph structure design and knowledge graph construction.

### Knowledge graph structure design

The purpose of designing a knowledge graph structure is to define the types of nodes and relationships to be included in the knowledge graph. The designed structure is the basis for knowledge extraction and the structural skeleton for knowledge graph construction [36]. Structural design is the key step constructing knowledge graphs for chronic diseases in elderly people. This paper used the seven-step ontology construction method developed by Stanford University to design the structure.

In existing knowledge graph research, there is a lack of theoretical guidance for knowledge graph structure design rules. The structure of the knowledge graph has some similarity with the ontology concepts. Ontology refers to a “formal, explicit and detailed description of a shared conceptual system”. Ontology defines the object types or concepts, their properties and relationships that exist in a specific domain. This is similar to how the knowledge graph concept layer uses nodes and relationships to describe the attributes of entities and the relationships between entities. The structure should not only cover the knowledge of all aspects related to care required by caregiver, but also avoid the overload of encyclopedic and excessive detailed and redundant information [37]

The seven-step ontology construction method developed by Stanford University involves determining the scope, considering reuse, enumerating terms, defining classes, defining attributes, defining constraints, and creating instances [38]. In this paper, we make some adjustments to the seven-step method, so that it can be more effective as a basis for the design of the knowledge graph schema layer.

According to the seven-step method, actual demand of the data, the knowledge graph (KG) definition of KGSCS is as followed.

*Definition 1. KG is a labeled, directed graph, which can be modeled as*

1 $$KG = (N, R, A, fN, fR)$$

Here, *N* represents the nodes of *KG*. The knowledge graph involved in this paper includes 31 types of nodes: name, sex, age, type, subsidy, income, expenditure, excretion, collateral chronic history, direct chronic history, sleep, diet, sport, physical condition, hobby, psychology, psychological factor, marriage, spouse, child, child type, caregiver, physical index, living environment, disease, medication, symptom, examination, smart phone, smart device and social.

The relationships *R* are divided into nine categories: basic information, physical information, diet information, initiation, prevention, treatment, diagnosis, complication and examination. These types of relationships connect the above nodes to form a knowledge graph model layer in line with the logic of chronic disease care knowledge retrieval.

**Fig. 4:**
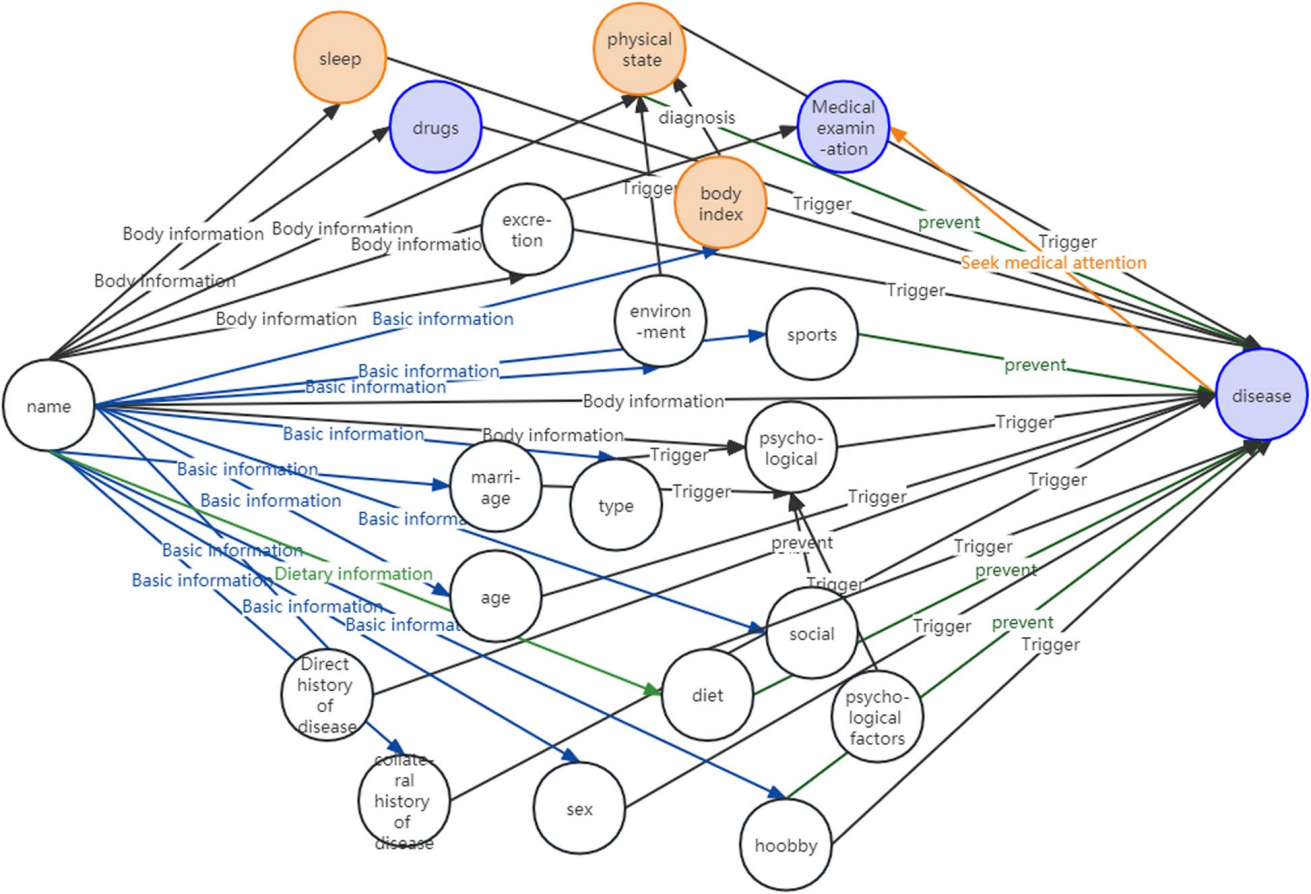
Knowledge graph structure design

*A* is the attributes set for *N*. Attributes are the characteristics that a node contains. Each node contains different attributes. *fN (fN ⊂ N* × *A)* describes what attribute *A* each node *N* contains. *fR (fR ⊂ (N*_*1*_*, N*_*2*_*)* × *R)* describes which two nodes of *N* is connected by relationship *R*. Therefore, *fN* and *fR* determine the storage format of the basic data file of the knowledge graph. The structure of the knowledge graph is shown in Fig. 4.

### Knowledge graph construction

This process extracts the "node-relationship-node" triplet from the processed text data according to the designed ontology structure. With the development of natural language processing, the task of entity relationship extraction combined with machine learning algorithms has become a major research focus in the field of knowledge graphs. Most of the triples in this paper were obtained through structured questionnaires and intelligent elderly care devices. Medical knowledge and nursing knowledge are extracted from nursing knowledge publications and expert knowledge through a combination of machine learning and manual verification. The knowledge extracted by the algorithm needs to be manually reviewed by experts before it can be used to construct knowledge graph. This is also an important step tin ensuring the reliability of knowledge graph.

The construction of the knowledge graph in this study was performed with Neo4j, which is a graph database software, specially used for graph data storage. The processed data were stored in a specified file format. The knowledge graph can be constructed by using neo4j import commands.

### Report generation and question answering

#### Report generation

Report generation is the primary means by which the system provides assistance to care staff who lack knowledge of care or are unfamiliar with elderly people's conditions. Based on the information in the system, the report generation module retrieves information according to basic information, physical health information, and dietary information, and generates a report on elderly care. Individuals can quickly gain a comprehensive understanding of the elderly's information and the care services that should be provided through this report. The detailed process of report generation is shown in the case study.

#### Question answering

The question answering module identifies the key words of the user's question through a machine learning algorithm. The corresponding knowledge was retrieved through the classification and understanding of the question and presented in the form of visualization.

**Fig. 5:**
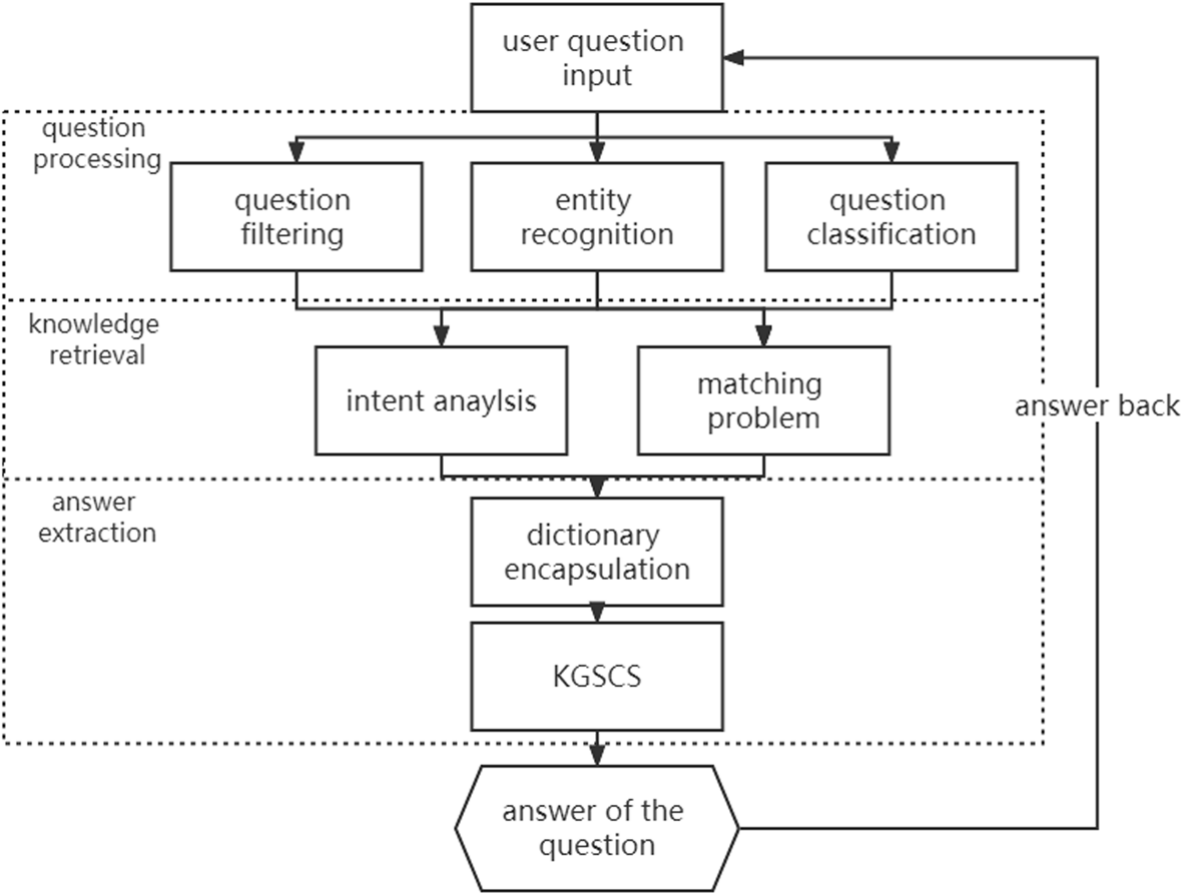
Question answering process

The whole process is in Fig. 5. Initially, it extracts feature words from medical data to construct a dictionary. For example, it may include terms like "disease names," "medications," and "symptoms." Subsequently, it builds a domain tree based on these features to filter domain-related keywords in user queries. For instance, if a user asks about a specific hospital department, the system can identify it by matching terms like "belong to what department" to the corresponding category in the domain tree.

Following this, the system identifies entities in user queries using the filtered feature word information. For example, if a user inquires about the cause of a disease, the system can recognize terms like "reason" or "why" as indicators of a query about disease etiology. It then categorizes the queries accordingly. For instance, queries related to known diseases and their treatments may be classified as "disease_drug" type questions.

Finally, the system converts user queries into a query language compatible with the smart care knowledge graph. An example formulation could be ["MATCH p = (m:Disease)-[r:TREATS]-(n:Medication) where m.name = 'hypertension' return p"]. This query language allows the system to retrieve relevant knowledge from the graph. For instance, it can use the query to find treatments for hypertension in the knowledge graph. Subsequently, the system returns the information to the user in natural language.

### KGSCS-based risk identification

Risk identification is one of the keys to chronic care for the elderly. This section will show how the system can prompt chronic disease risk. According to the actual situation and the experience in the research process, the potential risks are divided into the following three categories: (1) information registration risk, (2) care risk and (3) medical risk.

#### Information registration risk

During the actual questionnaire collection and work experience with caregivercaregiver, the content filled out by the elderly and their families may be wrong. There are many reasons for this phenomenon. Sometimes because the elderly and their families are advanced in age. Sometimes iit is a lack of knowledge. Sometimes it is psychological. Among these errors and omissions, past medical history, medication, and physical condition can lead to more serious consequences. In severe cases, it can be life-threatening. According to a 2014 survey by the China Food and Drug Administration, 2.5 million people in China suffer health damage and 200,000 people die each year because of incorrect medication. Although there are instructions on regular medicines, it takes expertise to use them correctly.

Smart care systems can solve this problem to some extent. The data can be verified by the other part of the knowledge graph through the data verification module. This module check input data based on certain rules. According to medical logic, there is a match between the drug, disease, and symptom. When a specific drug is used but the corresponding disease is not filled in, it often means that the elderly or their family members have made mistakes in filling in the information. Based on these inspection rules, the corresponding risk can be predicted. This process is similar to the logic of using knowledge graphs for aided disease diagnosis. For example if the elderly people antihypertensive drugs but do not register for hypertension, the module will pop up warning. This process can be represented as follows:

$$\begin{array}{c}\begin{array}{c}{\textit{fR}}_{\mathit1}\mathit\subset\mathit({\textit{N}}_\textit{name}\mathit,\mathit\;{\textit{N}}_\textit{drug}\mathit)\mathit\times{\textit{R}}_{basic\mathit\;information}\\{\textit{fR}}_{\mathit2}\mathit\subset\mathit({\textit{N}}_\textit{durg}\mathit,\mathit\;N_\textit{disease}\mathit)\mathit\times{\textit{R}}_\textit{cure}\\\begin{array}{c}{\textit{fR}}_{\mathit3}\mathit\subset\mathit({\textit{N}}_\textit{name}\mathit,\mathit\;{\textit{N}}_\textit{disease}\mathit)\mathit\times{\textit{R}}_{basic\mathit\;information}\\If\mathit\;{\textit{fR}}_{\mathit1}\mathit,\mathit\;fR_{\mathit2}\mathit=True\mathit\;and\mathit\;{\textit{fR}}_{\mathit3}\mathit=\textit{false}\end{array}\end{array}\\prompt\mathit\;risk\end{array}$$

The data validation process can also be extended to more general chronic disease risk monitoring. Identification of potential disease risks plays an important role in the generation of care reports.

#### Care risk

Care risk focuses on identifying health risks based on the daily living habits of the elderly. The network formed by nodes such as diet, sleep, psychological status, and exercise with diseases and symptoms. Because the pathogenesis and causes of chronic diseases in old age are complex, the best way to prevent them is to disclose all potential risk factors and pay more attention to them in daily care. This system can provide exclusive risk monitoring for each elderly person, rather than health knowledge in the sense of popular science.

Specifically, through the combination of personalized information about the elderly and the care knowledge map, a dedicated user-disease network is formed. A simple two-step, three-step search can identify risk factors for the lifestyle and physical conditions of the older person and indicate them in the report.

#### Medical risk

Medical risk can be more clearly identified through the path of medical knowledge risk. These risks are often more critical and important than care risks.

The first is the risk of physical conditions in the elderly based on sensor data. The blood pressure, weight and blood sugar levels of the elderly collected by smart sensors, and the sleep status collected by smart mattresses can be transformed according to medical standards. For example:

$$\begin{array}{c}\begin{array}{c}\begin{array}{c}{\textit{fR}}_{\mathit1}\mathit\subset\mathit({\textit{N}}_\textit{name}\mathit,\mathit\;{\textit{N}}_{blood\mathit\;pressure}\mathit)\mathit\times{\textit{R}}_{body\mathit\;information}\\{\textit{fR}}_{\mathit2}\mathit\subset\mathit({\textit{N}}_{blood\mathit\;pressure}\mathit,\mathit\;{\textit{N}}_\textit{hypertension}\mathit)\mathit\times{\textit{R}}_\textit{diagnosis}\\{\textit{fR}}_{\mathit3}\mathit\subset\mathit({\textit{N}}_\textit{name}\mathit,\mathit\;N_\textit{hypertension}\mathit)\mathit\times{\textit{R}}_\textit{diagnosis}\end{array}\\If\mathit\;{\textit{N}}_{blood\;pressure\mathit.\textit{SBP}}\mathit>\mathit=\mathit{140}mmHg\mathit\;or\mathit\;{\textit{N}}_{blood\mathit\;pressure\mathit.\textit{DBP}}\mathit>\mathit=\mathit{90}\textit{mmHg}\\{\textit{fR}}_{\mathit2}\mathit,\mathit\;{\textit{fR}}_{\mathit3}\mathit=\textit{Ture}\end{array}\\prompt\;risk\end{array}$$

The Table 1 illustrates the currently included sensors and their data transformation processes.

**Table 1:** Sensors and their data transformation processes

Sensors	Data	Standard*
Smart blood pressure monitor	Numeric, blood pressure	1st degree: SBP > = 130 mmHg/ DBP > = 80 mmHg; 2nd degree: SBP > = 140mmHg/ DBP > = 90mmHg;
Smart blood glucose meter	Numeric, blood sugar	Fasting blood glucose standard (mmol/L): Good: 4.4 ~ 6.1 Normal: 6.1 ~ 7.2 Fair: 7.2 ~ 8.2 Poor: 8.3 ~ 9.9 Extremely Poor: 10.0 ~
Smart scale	Numeric, weight	Underweight: BMI < 18.5 Normal weight: 18.5 ≤ BMI < 24.0 Overweight: 24.0 ≤ BMI < 28.0 Obesity: BMI ≥ 28.0
Smart mattress	Time series, bedridden status	Nighttime frequency, Prolonged lying time

^*^The above standards are formulated based on relevant medical guidelines in China

In addition, medical risk identification also includes the risk of interaction between drugs and the risk of interaction between diet and drugs. With the development of medical technology and the improvement of systematic knowledge, more medical risk identification rules will be gradually applied and reflected in the final personalized care guidelines.

## Results

### Case study of personalized elderly care guidelines

This paper takes 20 specific cases of elderly people who participated in a trial of the smart care system as an example to show how the system provides help and support for elderly chronic disease care services. In order to protect personal privacy, the specific information of the elderly was not provided.

Firstly, the elderly or their family members filled out the questionnaire with the help of caregivers, and smart devices such as blood pressure monitors, smart mattresses, and oximeters were used. Subsequently, the system uploaded the questionnaire and various body data collected by smart devices to the system. The system then processes the data into triples through a series of steps, which are linked with the knowledge graph. At this point, the system performs data validation, which is part of the risk identification function,

According to the association of drugs taken by the elderly, it can be known that the drugs taken by the elderly are the drugs for the treatment of diabetes, and the relevant disease information of the elderly is not registered. Therefore, caregiver should be reminded to confirm the medication and disease status of the elderly.

**Fig. 6:**
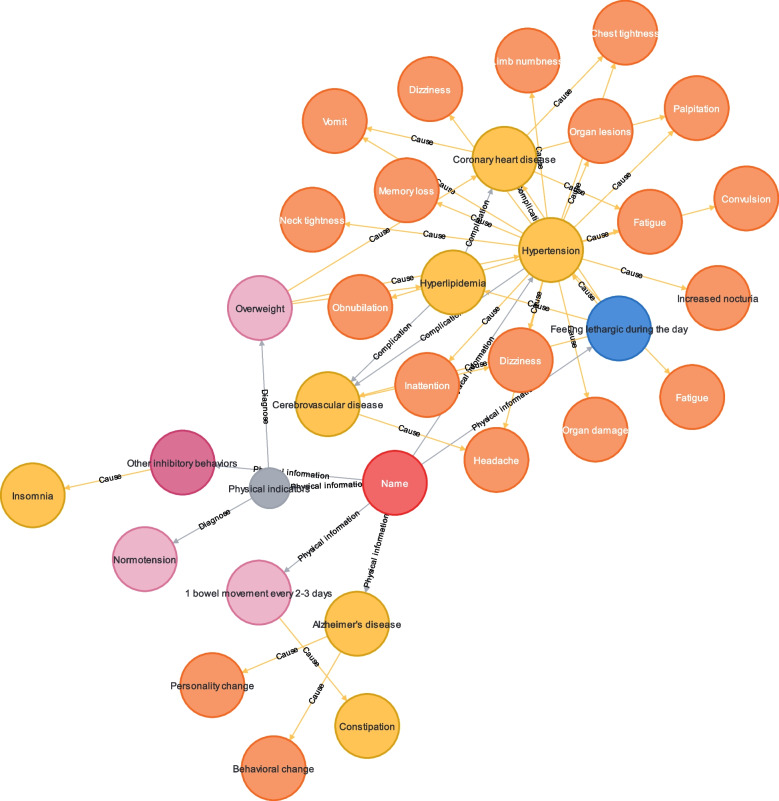
Case study

The full set of guidelines included 3 main parts, namely “basic information”, “health information” and “diet information”. Each section contains two subsections on risk identification and care recommendations. The purpose of these chapters is to search for the contents of relevant nodes and relationships according to a fixed retrieval mode, and to generate graphic guides through standardized expression. The following is a presentation of the risk factors for elderly individual's health care based on their health information (Fig. 6):

The physical indices of the elderly individuals showed that the participant was overweight and experiencing constipation, but the blood pressure was within normal range. Various bodily conditions are associated with risky diseases such as coronary heart disease, hypertension, hyperlipidemia, and cerebrovascular disease. Among these factors, coronary heart disease, hypertension, and hyperlipidemia are influenced by both body weight and psychological state. Therefore, it is important to pay attention to weight management and psychological well-being.

Alzheimer's disease may cause personality and behavioral changes. If such changes occur, caregivers should pay attention to the elderly individual's condition and course of Alzheimer's disease.

Coronary heart disease may cause symptoms such as vomiting, chest tightness, and fatigue. This individual's risk of coronary heart disease is due to complications from hypertension and sleep quality issues.

Hypertension can cause various symptoms and is a preexisting condition for the elderly individual. Hypertension can increase the risk of cerebrovascular and coronary heart diseases. Relevant care should be taken for patients with hypertension.

Hyperlipidemia can easily cause coronary heart disease and cerebrovascular disease, and it is a disease that elderly women are prone to. Additionally, poor sleep quality increases the risk of developing hyperlipidemia. Elderly people with high blood pressure, should pay attention to the diet to achieve low oil and low salt. The system will also give you the types of foods to try. Older adults did not provide information on blood pressure. It is very important to measure blood pressure in patients with hypertension, and blood pressure should be measured regularly. When symptoms of hypertension were observed, the patients were asked to the hospital to check for corresponding symptoms, and take the relevant drugs according to the doctor's advice.

### QA module and data update function

This system is capable of providing users with additional assistance beyond report generation. In daily care, the caregiver input: “What is better to eat for hypertension?”. The question–answer module retrieves relevant information from the system through transformation, displays the types of food eaten for hypertension matters in the form of a list, and provides visual interactive displays. Other aspects of hypertension prevention, such as daily living habits and hobbies, can be retrieved. When the elderly have regular examinations, doctors and other professionals can also conduct a more detailed search through this system, and configure more suitable combination therapy drugs for the elderly.

The elderly regularly uses wearable smart devices and smart mattresses to update the body data status to the server. Additionally, updates to the information in the questionnaire or medical-related knowledge can be performed through submission. The knowledge graph software Neo4j supports batch deletion and addition of relationships in the graph. The server updates the data and uploads the knowledge graph. After the system data update, the current risk of diseases in the elderly can be suggested according to the links between the elderly and diseases and symptoms, combined with the disease prediction model based on knowledge graph established by previous studies.

### System effectiveness evaluation

**Fig. 7:**
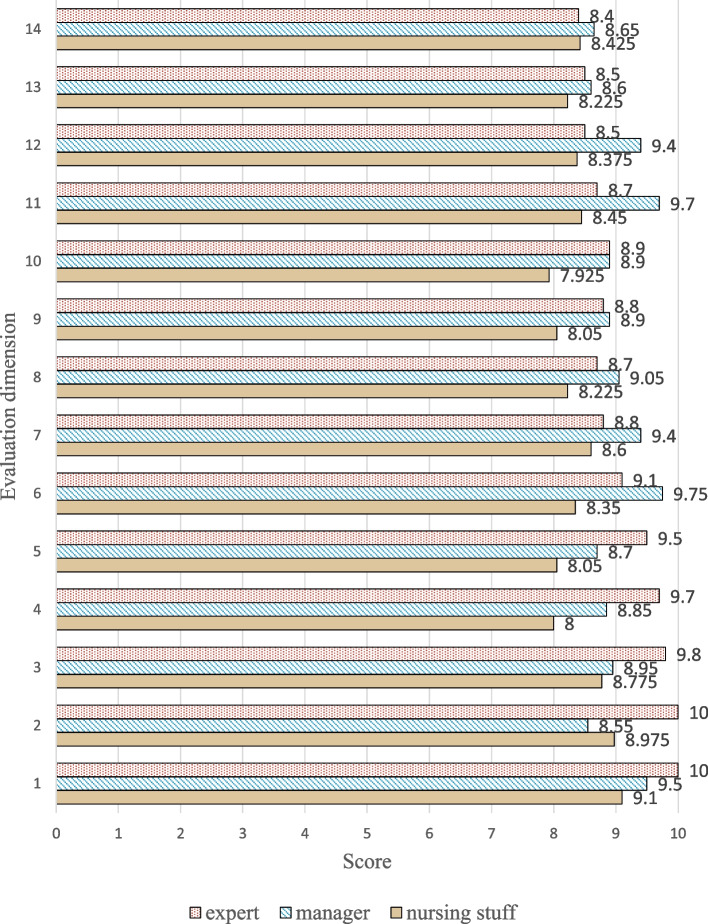
Expert evaluation results, the full score is 10

To validate the usefulness of the care reports generated by the system, we invited seven domain experts to score them. The panel of experts consisted of four professional caregivers, two care facility managers, and one medical specialist. The ten elderly individuals involved in this case study were the recipients of the services currently provided by the caregivers, hence they were very familiar with their conditions. Given this premise, we asked the experts to simulate a scenario in which they lacked the relevant professional skills and were unfamiliar with the elderly individuals' situations. Subsequently, the experts read the care reports generated by the system and evaluated how much care information and knowledge the reports were able to provide for each individual elderly (Table 2). Finally, we obtained an expert rating scale of 10 (Fig. 7).

**Table 2:** Indicators of evaluation

Chapter of the Care Report	Indicators of evaluation
Basic information	The extent to which the elderly basic information graph helps caregivers to have a clearer understanding of the overall basic information of the elderly
	The extent to which a clear understanding of the elderly's income and expenses helps in providing care management for the elderly
	The extent to which a clear understanding of the elderly's current illness, medical history, and family medical history helps in providing care management for the elderly
	The extent to which understanding the elderly's preferences, exercise habits, and social situation helps in providing care management for the elderly
	The extent to which the care risk analysis of basic information helps caregivers in care management
Health support	The extent to which the elderly's Physical health graph helps caregivers to have a clearer understanding of the overall physical health of the elderly
	The extent to which a clear understanding of the elderly's medication situation helps in providing care management for the elderly
	The extent to which a clear understanding of the elderly's behavior and clinical manifestations helps in providing care management for the elderly
	The extent to which the risk analysis chart for the elderly's physical health helps in providing care management for the elderly
	The extent to which the care advice chart for the elderly's physical health helps in providing care management for the elderly
Dietary information	The extent to which the dietary profile graph helps caregivers to have a clearer understanding of the elderly's nutritional status
	The extent to which a clear understanding of the elderly's dietary habits and behavior helps in providing care management for the elderly
	The extent to which the care risk chart for the elderly's diet information helps in providing care management for the elderly
	The extent to which the care advice for the elderly's diet helps in providing care management for the elderly

## Discussion

The following conclusions can be drawn from the expert scoring results. Overall, the experts believe that the system-generated reports are capable of providing considerable assistance to care services. Among them, because of their familiarity with the actual conditions of the elderly and their grasp of more details and information, caregivers rated the reports lower than managers and medical experts. Managers and medical experts are less familiar with the actual conditions of the elderly and thus rated the reports higher, considering them to provide comprehensive and rich information. For the three specific dimensions, the basic information dimension received the highest rating. The system's next direction for optimization is to add more rich content to the body health and diet information.

For comparison, this paper selected several knowledge graphs for comparison. The results showed that the KGSCS was prominent in terms of data source diversity and functional comprehensiveness. It can solve practical problems in care more effectively (Table 3).

**Table 3:** Functional comparison

	KGSCS	d-DC [34]	GENA [39]	MedKgConv [40]	smell and taste disorder KG [41]
Data source	Personal information; 2. Open source medical knowledge; 3. Smart devices data	1. Open source medical knowledge; 2. disease samples	1. Open source medical knowledge;	1. Open source medical knowledge;	1. Open source medical knowledge; 2. disease samples
Care knowledge	✔	✖	✔	✖	✖
Data verification	✔	✔	✖	✖	✖
Data updating	✔	✔	✔	✔	✔
Question answering	✔	✖	✖	✔	✔
Report generation	✔	✔ (disease classification)	✖	✖	✖

The Nielsen Norman Group, founded by usability experts Don Norman and Jakob Nielsen, is a prominent authority in the field of user experience (UX) design. They have outlined several basic principles that contribute to creating effective and satisfying user experiences. We evaluated the system based on the basic principles of User Experience (Nielsen Group).

1. Visibility of system status: The system provides clear feedback, allowing users to understand the status of caregiving tasks and alerting them to any changes or updates in the elderly person's condition. This ensures users can always be aware of the system's operational status, aligning with the principle.
2. Match between system and the real world: The system employs familiar language and terminology, making it easy for caregivers and elderly individuals to understand and operate. This design ensures that user interactions within the system match their experiences in the real world, adhering to the principle.
3. User control and freedom: Users have the autonomy to control caregiving tasks and can easily navigate the system to access relevant information or perform actions. This design ensures users have control over the system and flexibility in their interactions, aligning with the principle.
4. Consistency and standards: The system maintains consistency in design and functionality, ensuring similar actions lead to similar outcomes and users can predict system responses. This consistency reduces cognitive load and enables users to use the system more easily, adhering to the principle.
5. Error prevention: The system minimizes errors by providing clear guidance, prompts, and safeguards. This preventive design reduces the likelihood of user errors and enhances user experience, aligning with the principle.
6. Recognition rather than recall: The system prioritizes visibility of options and actions, reducing the need for users to remember specific details from previous interactions. This reduces cognitive burden and improves system usability, adhering to the principle.
7. Flexibility and efficiency of use: The system caters to users with varying levels of experience and expertise, offering shortcuts for experienced users while remaining accessible to novices. This flexibility improves system efficiency and user satisfaction, aligning with the principle.
8. Aesthetic and minimalist design: The system features a clean and intuitive interface, focusing on essential information and minimizing clutter. This enhances usability and user satisfaction, adhering to the principle.
9. Help users recognize, diagnose, and recover from errors: The system provides clear error messages and prompts to guide users in troubleshooting issues and recovering from mistakes effectively. This ensures users can easily identify and resolve errors, reducing frustration, and aligning with the principle.
10. Help and documentation: While the system aims to be intuitive and easy to use without extensive documentation, it still provides help resources and guides for users who require additional assistance. This design ensures users can receive support and guidance when needed, adhering to the principle.

In general, the intelligent care system can effectively remind caregiver to adjust the content of care services and improve the quality of life and physical health of the elderly. This makes it possible for nonprofessional general caregiver or the children and grandchildren of the elderly to be competent for nursing work, and reduces the difficulty of replacing caregiver.

## Limitations

In today’s information-rich environment, NLP algorithms and ML methods offer significant advantages for processing large-scale text and data. Recognizing their potential, we aim to incorporate adjusted or improved versions of these algorithms into our system framework for future research. This will enhance our ability to analyze complex medical data and contribute to advancing healthcare understanding and improvement. Through ongoing refinement of our techniques, we anticipate achieving significant outcomes in healthcare development.

Furthermore, our future outlook involves expanding our system’s usage scope and gathering additional data. We plan to collaborate further with healthcare professionals and institutions to validate and extend the implementation of our system across diverse caregiving environments. By persisting in our commitment to innovation and collaboration, we aim to continue making significant strides in the field of elderly smart caregiving. Ultimately, our goal remains centered on enhancing the quality of life for elderly individuals and their caregivers through the advancement of our system's capabilities and the accumulation of comprehensive data insights.

## Conclusion

With the increasing aging of the global population, it is important for society to establish a long-term care system. With the rapid development of Internet information technology and the rapid rise of artificial intelligence technology, traditional care services have been transformed from artificial to intelligent. These technologies continue to promote the extension of care services in content, changes in methods and functional breakthroughs to provide better services for the elderly. At present, smart devices and medical knowledge graphs have broad application prospects, and the smart system that can integrate the two and be applied to the intelligent care of the elderly has research value.

The knowledge graph of chronic diseases for the elderly is an important work in the construction of intelligent care system. The traditional medical disease knowledge graph cannot meet the needs of chronic disease care and nursing knowledge for the elderly. Therefore, it is necessary to establish the chronic disease care knowledge graph for the elderly. In this paper, smart devices data, personal information questionnaires and open source chronic disease care knowledge for the elderly were used to construct the knowledge graph. The premise of constructing knowledge graph is to design the structure of knowledge graph well. With reference to ontology design methods such as the seven-step method, high-quality knowledge graph structures can be built. The data verification module can check the accuracy of the filled information according to the knowledge graph.

To help caregivers who lack professional nursing knowledge or are unfamiliar with the elderly to provide care services for the elderly more quickly and professionally, this paper designed a care report generation module. The module retrieves comprehensive information about a specific elderly and generate care reports according to a certain structure. This paper also designed a question and answer module, which was used to convert the user's general questions into query statements and realize the real-time convenient human–machine question and answer function.

In the future, knowledge graph can also be combined with risk early warning model to provide new variables for risk early warning model. These variables depend on the unique graph structure of knowledge graph, such as the number and length of paths associated with nodes, which can effectively supplement the predictive factors of risk early warning model and improve the accuracy of early warning systems.

### Supplementary Information

**Supplementary Material 1.**

## Data Availability

The data that support the findings of this study are available from Beijing Academy of Science and Technology, Research Institute for Smart Aging but restrictions apply to the availability of these data, which were used under license for the current study, and so are not publicly available. Data are however available from the authors upon reasonable request and with permission of Beijing Academy of Science and Technology, Research Institute for Smart Aging.

## Abbreviation

KGSCS: Knowledge graph smart care system
Smote-CT: Systematized Nomenclature of Medicine – Clinical Terms
UMLS: Unified Medical Language System
